# Relationship between subjective well-being and aripiprazole: an [^11^C]raclopride PET study

**DOI:** 10.1038/s41598-022-16130-5

**Published:** 2022-07-15

**Authors:** Seoyoung Kim, Elena Younhye Ock, Jun Soo Kwon, Euitae Kim

**Affiliations:** 1 Department of Psychiatry, Seoul National University Bundang Hospital, 82, Gumi-ro 173beon-gil, Bundang-gu, Seongnam-si, Gyeonggi-do 13620 Republic of Korea; 2 Interfaculty Program Cognitive Science, McGill University, Montreal, QC Canada; 3 Department of Psychiatry, College of Medicine, Seoul National University, Seoul, Republic of Korea; 4 Department of Brain and Cognitive Sciences, College of Natural Sciences, Seoul National University, Seoul, Republic of Korea

**Keywords:** Molecular neuroscience, Schizophrenia

## Abstract

The dopamine blockade by antipsychotics trigger subjective dysphoria. Compared with D2 antagonists, aripiprazole, a D2 partial agonist, was expected to produce a different experience. Indeed, a previous study reported no relationship between the D2 receptor occupancy by aripiprazole and subjective dysphoria, while the D2 receptor occupancy by antagonists was associated with negative subjective experiences. This study revisited the relationship in patients treated with aripiprazole by using an inhibitory E_max_ model, which enables the individual drug-free binding potential and D2 receptor occupancy to be properly estimated. Eight patients with schizophrenia who have been clinically stable on aripiprazole were enrolled. Assessments including Positive and Negative Syndrome Scale (PANSS) and Subjective Well-being under Neuroleptics Scale (Kv-SWN) were administered. [^11^C]raclopride PET scan were conducted 2, 26, and 74 h after aripiprazole administration. Regression analysis showed a significant negative association between the D2 receptor occupancy by aripiprazole in the striatum and the Kv-SWN (R^2^ = 0.55, p = 0.036), but the PANSS total score was not associated with the Kv-SWN (R^2^ = 0.42, p = 0.080). The negative association between D2 receptor occupancy by aripiprazole and subjective well-being implies that clinicians should find the lowest effective doses of aripiprazole for clinically stable patients to improve their subjective experiences and clinical outcomes.

## Introduction

Negative subjective experience of patients taking antipsychotic drugs is a crucial predictor of poor adherence, leading to psychotic relapse with antipsychotic discontinuation^1,2^. Neuroleptic dysphoria refers to subjective and subtle side-effects by antipsychotic drugs including unpleasant mood, affective blunting, low motivation, and an inability to engage in pleasant-evoking behavior^3,4^. Patients’ subjective reports on neuroleptic dysphoria, however, had long been consistently ignored, as psychiatrists focused on addressing more tangible physical side-effects, such as extrapyramidal and autonomic symptoms^1,3^. With the development of atypical antipsychotic drugs that cause fewer extrapyramidal symptoms and other physical side-effects^5^, the clinical relevance of subjective dysphoric feeling induced by antipsychotic drugs has drawn more attention^6^. Furthermore, Naber et al.^7^ showed that subjective well-being of patients treated with antipsychotic drugs was not reliably predicted by changes in psychopathology. This suggests that clinicians and patients might evaluate the outcome of antipsychotic treatment differently. Thus, patients’ dysphoric feeling induced by antipsychotic drugs must be better understood to ensure treatment adherence and good clinical outcome^3^.

Molecular imaging studies have revealed that the neurobiological mechanism underlying neuroleptic dysphoria is associated with altered dopamine functioning after antipsychotic treatments^8–10^. Indeed, the inverse relationships between striatal dopamine D2 receptor occupancy by antipsychotic drugs and subjective well-being were demonstrated in patients^8,10–12^. A striatal D2 receptor occupancy of 60–70% was optimal in terms of subjective experience^9,11,13^. However, the relationship between the D2 receptor occupancy by antipsychotic drugs and neuroleptic dysphoria may differ according to their pharmacological profile. For example, antipsychotic drugs binding tightly to D2 receptors exhibited inverse relationships between the occupancy and negative subjective feelings; loose-binding antipsychotic drugs did not^14^. Moreover, dopamine antagonists and a partial agonist like aripiprazole differed in this regard^10^.

Aripiprazole has been the focus of much clinical attention due to its unique receptor profile as a dopamine partial agonist^15^. Though aripiprazole acts as an antagonist in circumstances of high dopamine receptor stimulation, it still retains some intrinsic activity as an agonist, making antagonism-related adverse effects less likely^16^. For example, Yokoi et al.^17^ showed that extrapyramidal side effects were not observed, regardless of whether striatal D2 receptor occupancy by aripiprazole exceeded 90%. Clinically, extrapyramidal symptoms are uncommon (less than 10–11%) in patients taking aripiprazole, and the drug has been shown to reduce prolactin levels, which is contrary to D2 antagonists^18,19^. Based on the results, aripiprazole was expected to produce a different subjective experience compared to antipsychotic drugs with D2 antagonism. Indeed, Mizrahi et al.^10^ demonstrated that patients with schizophrenia who switched from D2 antagonists like olanzapine and risperidone to aripiprazole have reported significant improvement in their subjective well-being, despite very high striatal D2 receptor occupancy over 80%. Moreover, no significant relationship between the D2 receptor occupancy by aripiprazole and patients’ dysphoric symptoms was observed, while higher D2 receptor occupancy by D2 antagonists was associated with negative subjective experience^10^. This may have suggested that aripiprazole having intrinsic dopamine activity may trigger less neuroleptic dysphoria regardless of its doses.

However, in the study by Mizrahi et al.^10^, the range of the D2 receptor occupancy by aripiprazole was narrow (82–95%), rendering it difficult to detect any relationship between the occupancy and the subjective well-being score; in contrast, the subjective well-being score ranged widely in that study [65–115] on Subjective Well-Being under Neuroleptics Scale (SWN)]^7^. Another methodological limitation was that receptor occupancies were calculated using the drug-free binding potentials of unrelated healthy controls. Although such substitution have been widely employed to calculate D2 receptor occupancy by antipsychotic drugs in patients with schizophrenia, the occupancy calculated by using the binding potentials from healthy controls could be biased because the disease has been reported to alter the binding potentials^20^. Without taking into account population differences in drug-free binding potential, true receptor occupancies may be under- or overestimated in patients currently taking antipsychotic drugs^21^. The limitations above may have made it challenging to explore the relationship between the occupancy by aripiprazole and neuroleptic dysphoria in the study by Mizrahi et al.^10^.

The abovementioned methodological limitations can be overcome by adopting the previously developed inhibitory E_max_ model, which is a validated method to estimate drug-free binding potentials in patients currently on psychotropic drugs^22^. It enables to investigate receptor binding potentials during treatment while disregarding the effect of the antipsychotic drug by employing nonlinear mixed-effects modeling with individual serial binding potentials^22^.

This study aimed to investigate the relationship between the D2 receptor occupancy by aripiprazole and neuroleptic dysphoria. As mentioned above, any relationship will be revealed when D2 receptor occupancy by aripiprazole varies widely and is accurately estimated. Thus, we sought to determine the relationship in patients treated with different doses of aripiprazole by using an inhibitory E_max_ model, which enables the individual drug-free binding potential and D2 receptor occupancy to be properly estimated in patients currently treated with psychotropic drugs^21^.

## Results

A total of eight patients, including six females and two males, participated in the study. Table 1 shows demographic and clinical characteristics of the patients. The mean (± SD) age, height, and weight of patients were 32.1 ± 9.7 years, 162.8 ± 10.4 cm, and 63.2 ± 16.3 kg, respectively. The mean (± SD) maintenance dose of aripiprazole prescribed for patients was 13.1 ± 11.6 mg (2 mg for one patient, 2.5 mg for one patient, 5 mg for two patients, 10 mg for one patient, 25 mg for two patients, and 30 mg for one patient). The mean (± SD) period for the maintenance dose was 25.1 ± 27.2 months. There was no concomitant medication prescribed for patients in the study.

**Table 1:** Demographic and clinical characteristics of patients.

**Demographic characteristics**		
Age (years)	32.1	(9.7)
Female gender (n)	6	(75.0)
Height (cm)	162.8	(10.4)
Weight (kg)	63.2	(16.3)
**Clinical characteristics**		
Aripiprazole dose (mg)	13.1	(11.6)
Duration of maintenance dose (month)	25.1	(27.2)
**PANSS score**		
Total	42.5	(8.9)
Positive symptoms	8.0	(1.1)
Negative symptoms	12.8	(4.9)
General symptoms	21.9	(3.8)
Kv-SWN score	93.0	(15.0)
SAS score	1.1	(0.9)
AIMS score	0.4	(0.7)
BARS score	0.7	(1.2)

All variables are presented as mean (± SD), or n (%).

*PANSS* Positive and Negative Syndrome Scale, *Kv-SWN* Korean version of Subjective Well-Being under Neuroleptics Scale-Short form, *SAS* Simpson–Angus Scale, *AIMS* Abnormal Involuntary Movement Scale, *BARS* Barnes Akathisia Rating Scale.

The average total scores (± SD) of positive and negative syndrome scale (PANSS) and the Korean version of subjective well-being under neuroleptics scale-short form (Kv-SWN) were 42.5 ± 8.9 and 93.0 ± 15.0, respectively. Aripiprazole did not induce significant extrapyramidal symptoms as revealed by the scores (mean ± SD) of the Simpson–Angus scale (SAS) (1.1 ± 0.9), the abnormal involuntary movement scale (AIMS) (0.4 ± 0.7), and the Barnes akathisia rating scale (BARS) (0.7 ± 1.2).

The average plasma concentrations (± SD) of aripiprazole were 335.4 ± 395.3 ng/ml, 257.7 ± 332.1 ng/ml, and 152.5 ± 217.3 ng/ml at 2, 26, and 74 h after aripiprazole administration. The mean binding potential (BP_ND_) (± SD) in the striatum measured at the corresponding time were 0.5 ± 0.1, 0.6 ± 0.2, and 0.7 ± 0.2, respectively. The average drug-free BP_ND_ (± SD) estimate from the inhibitory E_max_ model was 1.5 ± 0.03. The mean D2 receptor occupancy (± SD) by aripiprazole calculated with individual drug-free BP_ND_ at 2 h after aripiprazole administration, right after the clinical assessments, was 53.0 ± 11.7%.

**Figure 1:**
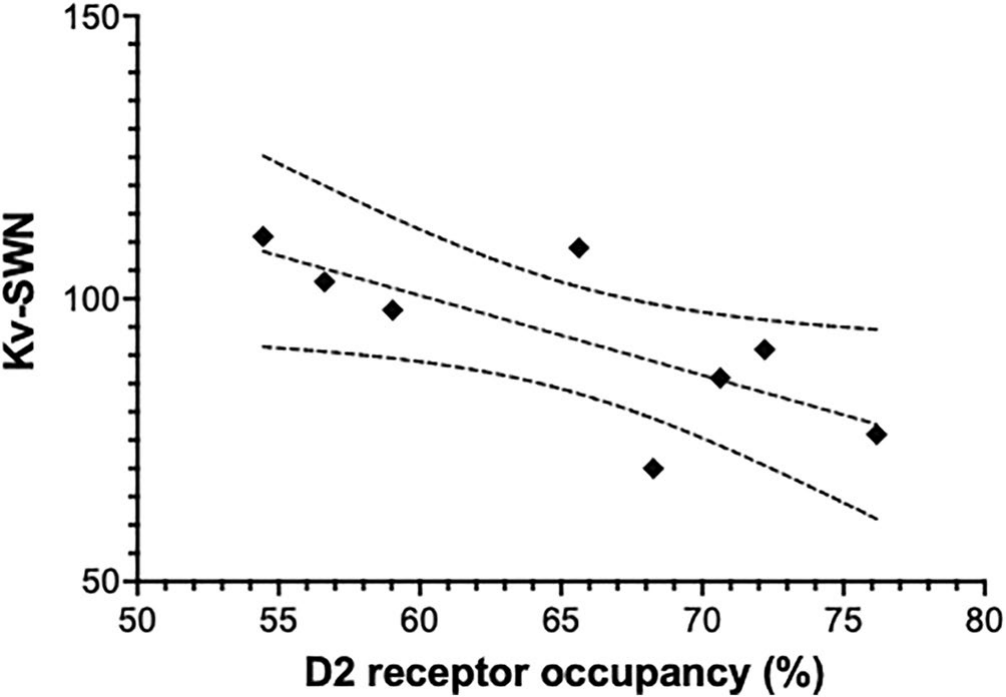
The relationship between D2 receptor occupancy by aripiprazole and scores of Korean version of subjective well-being under Neuroleptics Scale-Short form (Kv-SWN) (R^2^ = 0.55, p = 0.036).

Regression analysis showed a significant negative association between the D2 receptor occupancy by aripiprazole in the striatum and the Kv-SWN (R^2^ = 0.55, p = 0.036), but the PANSS total score was not associated with the Kv-SWN (R^2^ = 0.42, p = 0.080). In the regression analysis for the relationship between the occupancy and Kv-SWN, the maximal cook’s distance was 0.2 (Figure 1).

## Discussion

The present study investigated the relationship between D2 receptor occupancy by aripiprazole and the subjective well-being of patients. For this, we enrolled patients who were clinically stable and receiving varying doses of aripiprazole. Furthermore, we estimated the individual D2 receptor occupancy by aripiprazole using the inhibitory E_max_ model^21,23,24^ and serial BP_ND_ data obtained at different time points. Our main findings are that lower D2 receptor occupancy by aripiprazole was related to a higher score of Kv-SWN; however, the Kv-SWN score was not significantly associated with the PANSS score. These results indicate that D2 receptor occupancy by aripiprazole may negatively affect subjective well-being and suggest that minimal effective doses of aripiprazole should, thus, be prescribed in clinically stable patients with schizophrenia for their better subjective experience.

Patients’ subjective well-being could have been affected by their psychopathology, as previously reported^7^. In the present study, the mean PANSS total score was 42.5, which can be considered as in remission in the aspect of symptom severity^25^. Furthermore, the statistical analysis showed no significant relationship between the psychopathology assessed by using PANSS and subjective well-being. Thus, the subjective well-being or neuroleptic dysphoria assessed in the present study was unlikely to be influenced by psychopathology. Meanwhile, extrapyramidal symptoms could have affected subjective well-being. However, as seen in the scores of SAS, AIMS, and BARS (Table 1), there were no significant extrapyramidal symptoms induced by aripiprazole, and this is not likely to be the case.

The antipsychotic efficacy of aripiprazole and its favorable safety and tolerability have been suggested to arise from the unique receptor profile of D2 partial agonism^16,26^. The D2 partial agonism of aripiprazole has also been expected to improve functional impairment leading to satisfactory quality of life^27^. Indeed, aripiprazole was reported to ameliorate cognitive impairment like working memory^23^, which is closely related to real-world functioning in schizophrenia^28^. In the aspect of quality of life, aripiprazole showed better efficacy compared with D2 receptor antagonists like paliperidone^29,30^.

The favorable tolerability of aripiprazole and its positive effects on functioning and quality of life led to the expectation that aripiprazole would produce a different subjective experience profile, compared with the conventional antipsychotic drugs with D2 antagonism. As mentioned above, Mizrahi et al.^10^ reported no significant relationship between the D2 receptor occupancy by aripiprazole and patients’ subjective well-being, while the D2 receptor occupancy by antagonists including olanzapine and risperidone was negatively correlated with the subjective well-being^8,10^. However, the reported different effects between aripiprazole and D2 antagonists on subjective well-being were compromised by some methodological issues described above.

We found that aripiprazole was also associated with a negative relationship between D2 receptor occupancy and subjective well-being. Partial agonists like aripiprazole can behave as an agonist or as an antagonist depending on the local dopamine concentration^27^. Neuroleptic dysphoria is presumably associated with the blockade of dopaminergic neurotransmission in the striatum. Indeed, the neuroleptic dysphoria was significantly correlated with D2 receptor occupancy by antipsychotic drugs in the striatum^12^. In addition, striatal dopamine depletion induced by alpha-methyl-para-tyrosine triggered dysphoric symptoms^31^. Though aripiprazole has intrinsic activity at the D2 receptor, it can act as an antagonist in the striatum, where dopamine activity has been reported to increase in schizophrenia^32^. The blockade of dopaminergic neurotransmission by aripiprazole in the striatum can induce neuroleptic dysphoria, as in the case of antipsychotic drugs with D2 antagonism.

Patients treated with aripiprazole exhibited a wide range of subjective well-being level in terms of Kv-SWN score, despite their mild clinical symptoms. Furthermore, the symptomatic severity was not related to subjective well-being, which is in line with the findings of previous study^7^. Since adverse subjective well-being of patients on antipsychotic treatment can compromise their adherence leading to the discontinuation of the treatment and psychotic relapse^1,2^, our finding urges clinicians to find the lowest dose of aripiprazole in order to minimize the dysphoric experience of patients for better subjective well-being.

With the application of the inhibitory Emax model, the individual D2 receptor occupancy by aripiprazole was successfully calculated in patients currently taking the medication. Moreover, the range of the estimated D2 receptor occupancy by aripiprazole in this study was much broader compared to the previous study by Mizrahi et al.^10^. Accordingly, we were able to see the relationship between D2 receptor occupancy by aripiprazole and subjective dysphoria, which explicitly showed negative correlation.

This study has several limitations to be taken into consideration when interpreting the results. First, we measured the D2 receptor occupancy by aripiprazole and related it to the subjective well-being. Though aripiprazole has a high affinity for the D2 receptor, it also has appreciable affinities for serotonin 5-HT receptors and behaves as a partial 5-HT1A agonist and a 5-HT2A antagonist^33^. The 5-HT system modulates a broad spectrum of brain functions, including mood, anxiety, and cognition^34^. We did not measure the pharmacological effect of aripiprazole on the 5-HT system. Thus, it is required to investigate the potential role of aripiprazole on the 5-HT system and its relationship with subjective well-being. Second, we recruited clinically stable patients who had been treated with aripiprazole for a considerable duration. It is a well-documented phenomenon that agonists can induce the internalization of D2 receptors^35,36^. Aripiprazole binding to D2 receptors with intrinsic activity could have induced the internalization of D2 receptors. In fact, the mean (± SD) drug-free BP_ND_ (1.5 ± 0.03) estimated in the present study was lower than the average (± SD) BP_ND_ (2.8 ± 0.3) measured in healthy controls^37^. This may reflect aripiprazole-induced internalization of D2 receptor. The internalization of the D2 receptors can affect the binding affinity of [^11^C]raclopride for D2 receptors^38^ and could have influenced the estimated D2 receptor occupancy by aripiprazole. Meanwhile, the internalization of D2 receptor is well accepted as a process of desensitization to dopamine stimuli. For instance, in vivo experiment demonstrated that D2 receptors was downregulated in response to drug abuse^39,40^. The desensitization could have been associated with the subjective well-being of patients treated with aripiprazole. However, this still remains unclear, and the present study, evaluating aripiprazole alone, cannot make a conclusion about this issue. This warrants a future study comparing a D2 partial agonist like aripiprazole with D2 antagonists.

## Conclusion

The D2 receptor occupancy by aripiprazole was negatively related to subjective well-being, implying that clinicians should find the lowest effective dose of aripiprazole for clinically stable patients to improve their better subjective experiences and clinical outcomes.

## Methods

The present study was approved by the Institutional Review Board of Seoul National University Hospital, Seoul, Korea, and was conducted in accordance with the Helsinki Declaration of 1975, as revised in 2008.

### Subjects

Eight right-handed, non-smoking patients with schizophrenia participated in the study. Seven of them were also enrolled in another study, as described in the previous report^23^. For the enrollment, patients with schizophrenia were required to have been treated with aripiprazole for at least 6 weeks which are expected for stable therapeutic effects of aripiprazole^26^ and to be clinically stable in this period determined with a total score of < 60 in the PANSS^25^.

After complete description of the study to the subjects, written informed consent was obtained. Screening tests included physical examinations, vital signs, laboratory tests (hematology, blood chemistry, and urinalysis), and a 12-lead electrocardiogram. A psychiatric interview with the Structured Clinical Interview for DSM-IV-TR Axis I Disorders, Research Version (SCID-I/P) was conducted^41^. Any subject with a medically significant abnormality and/or a psychiatric disease other than schizophrenia was excluded.

### Study design

**Figure 2:**
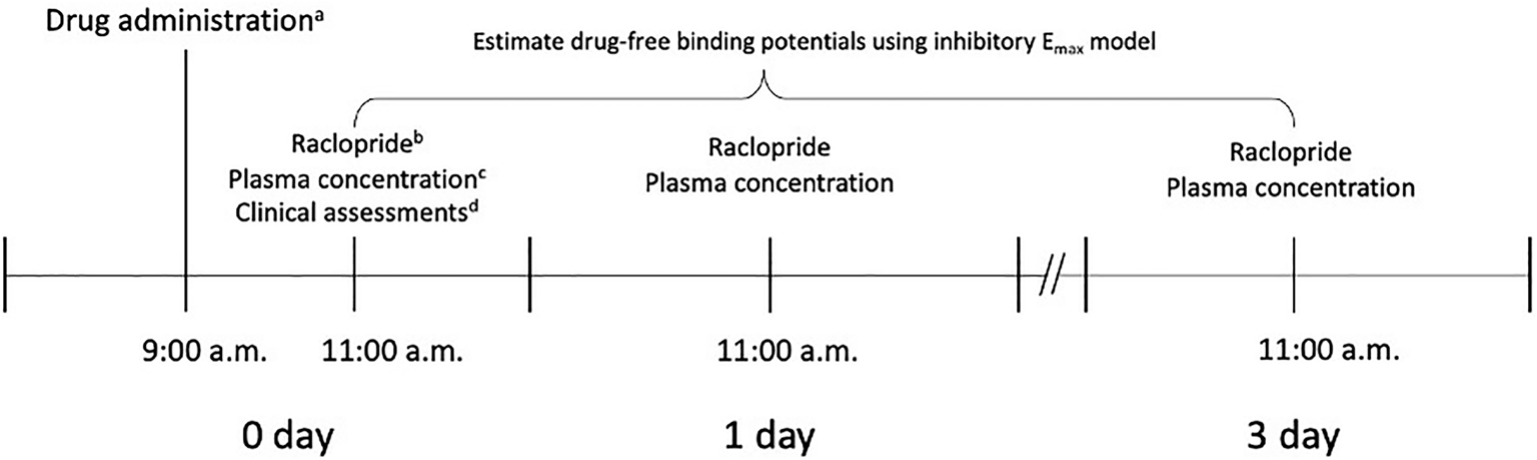
Diagram illustrating study protocol. (**a**) Aripiprazole; (**b**) positron emission tomography scan with [^11^C]raclopride; (**c**) plasma concentration of aripiprazole; (**d**) clinical assessments with Positive and Negative Syndrome scale, Korean version of subjective well-being under Neuroleptics Scale-Short form, Simpson-Angus Scale, Abnormal Involuntary Movement Scale, and Barnes Akathisia Rating Scale.

Figure 2 illustrates the study design. Subjects were required to stay at the Clinical Trial Center, Seoul National University Hospital, for serial positron emission tomography (PET) scans with [^11^C]raclopride. They were instructed to abstain from caffeine or caffeine-containing products (e.g., coffee, coke, black tea, green tea, and chocolate), grapefruit-containing products, alcohol, and smoking for the duration of study

After fasting for at least 4 h, the subjects received the same oral dose of aripiprazole as they had been regularly prescribed, with 240 mL of water, at 9:00 a.m. Serial PET scans with [^11^C]raclopride were conducted 2, 26, and 74 h after administration of a single dose of aripiprazole. Blood samples for the determination of plasma aripiprazole concentration were obtained within 5 min before the PET scans with no additional dosing of aripiprazole during the study period.

The neuroleptic dysphoria related with aripiprazole was assessed with Kv-SWN^7,42^ and psychotic symptoms in the participants were examined by using PANSS. The extrapyramidal symptoms by aripiprazole were evaluated using the SAS^43^, AIMS^44^, and BARS^45^. The assessments were preformed just before the first PET scan with [^11^C]raclopride.

### Positron emission tomography and image analysis

The PET images were obtained as previously described ^23^. All PET scans were performed on an ECAT EXACT 47 scanner (full-width half-maximum [FWHM] = 4.6 mm) (Siemens-CTI, Knoxville, TN, USA). Before the acquisition of the dynamic scan, a transmission scan was performed using three Ge-68 rod sources for attenuation correction. Dynamic 3D emission scans over 60 min (15 s × 8, 30 s × 16, 60 s × 10, and 240 s × 10 frames) were conducted after a bolus injection of 370–740 mBq [^11^C]raclopride. The data from the dynamic scans were reconstructed in a 128 × 128 × 47 matrix with a pixel size of 2.1 × 2.1 × 3.4 mm by means of a filtered back-projection algorithm, employing a Shepp–Logan filter, with a cutoff frequency of 0.3 cycles/pixel.

Static PET images, produced by combining all the frames of dynamic images, were co-registered with the magnetic resonance (MR) images of the same individual obtained on a GE Sigma 1.5 T scanner. The MR images were used to define the regions of interest (ROIs) including the striatum and the reference region (the cerebellum)^46^. The ROIs were drawn on the subject’s T1 MR images by a single rater on ten axial slices for the striatum and cerebellum. We used the transformation parameters obtained from the co-registration of the static PET and MR images with SPM8 and transferred the ROI onto the dynamic PET images to access the time–activity curves for the whole volume of interest by applying the transformation parameters.

### D2 receptor occupancy by aripiprazole

Three BP_ND_ in the striatum obtained from each patient were calculated by using a simplified reference tissue model^47,48^. The D2 receptor occupancy by aripiprazole was defined as the percentage reduction of BP_ND_ with aripiprazole treatment, compared with drug-free condition as follows:

1 $$Occupancy\left( \% \right) = \frac{{BP_{{ND_{drug - free } }} - BP_{{ND_{drug} }} }}{{BP_{{ND_{drug - free } }} }} \times 100,$$

where $BP_{{ND_{drug - free } }}$ is the BP_ND_ when D2 receptor is not occupied by aripiprazole and $BP_{{ND_{drug } }}$ is the BP_ND_ obtained after the administration of aripiprazole.

As patients enrolled for the present study were currently taking aripiprazole, we obtained the drug-free BP_ND_ where aripiprazole effects were removed by using an inhibitory E_max_ model in Eq. (2) with individual serial BP_ND_ data as previously described and validated^21,23^.

2 $$BP_{ND} = BP_{{ND_{drug - free} }} - \frac{{I_{\max } \times Conc^{r} }}{{IC^{r}_{50} + Conc^{r} }},$$

where I_max_ is the maximum inhibitory effect, Conc is plasma concentration of aripiprazole, IC_50_ is the plasma concentration associated with a 50% decrease of BP_ND_, and r is the Hill coefficient. When a very high dose of aripiprazole is administered, BP_ND_ is equal to zero, and it follows from Eq. (2) that I_max_ is equal to drug-free BP_ND_.

Nonlinear mixed-effects modeling simultaneously estimates fixed effects and random effects in the inhibitory E_max_ model. Fixed effects are parameters, including I_max_, IC_50_, and r which describe the relationship between the plasma aripiprazole concentration and BP_ND_ in the population. The random effects are composed of inter-individual variability and residual variability.

From the nonlinear mixed-effects modeling, we obtained individual estimates of drug-free BP_ND_ as follows:

3 $${\text{Drug - free}}\,{\text{BP}}_{{{\text{NDi}}}} = {\text{I}}_{{{\text{max}}}} \cdot {\text{exp}}\left( {\eta_{{\text{i}}} \,{\text{of}}\,{\text{I}}_{{{\text{max}}}} } \right),$$

where drug-free BP_NDi_ indicates the true drug-free BP_ND_ value for the ith individual, I_max_ is typical population value of the maximum inhibitory effect, and η_i_ is inter-individual variability of the maximum inhibitory effect for ith individual. The estimations were conducted using NONMEM version 7.2.0. software (GloboMax, Ellicott City, MD, USA).

### Statistical analysis

Descriptive analysis was conductive for demographic data. Regression analysis was employed to investigate the relationship of D2 receptor occupancy by aripiprazole with Kv-SWN and PANSS scores. The effects of individual data points on the regression were evaluated using the Cook’s distance test to detect outliers. The statistical analysis was performed using SPSS version 27.0.1.0. software (IBM, Armonk, NY, USA).

## Data Availability

The datasets generated during and/or analysed during the current study are available from the corresponding author on reasonable request.
